# Artesunate: The Best Drug in the Treatment of Severe and Complicated Malaria

**DOI:** 10.3390/ph3072322

**Published:** 2010-07-21

**Authors:** Qigui Li, Peter Weina

**Affiliations:** Department of Drug Discovery, Division of Experimental Therapeutics, Walter Reed Army Institute of Research, 503 Robert Grant Avenue, Silver Spring, MD 20910-7500, USA; E-Mail: peter.weina@us.army.mil

**Keywords:** artesunate, artemether, quinine, quinidine, severe malaria, complicated malaria

## Abstract

This review summarizes progress in treating severe and complicated malaria, which are global problems, claiming at least one million lives annually, and have been accompanied by advances in our understanding of the pathogenesis of severe malaria complications. New drugs such as intravenous artesunate (AS) and intramuscular artemether (AM) are improving outcomes and decreasing malaria deaths. Trials comparing AM to the traditional parenteral drug, quinine, have not demonstrated however convincing evidence of a mortality advantage for AM. The South East Asian Quinine Artesunate Malaria Trials (SEAQUAMAT), a multicenter, randomized, open-label study comparing AS with quinine showed that parenteral AS was shown to be associated with a 35% reduction in the risk of mortality compare to quinine, and is now the recommended treatment by the WHO for severe and complicated malaria in low-transmission areas and in the second and third trimesters of pregnancy, with almost all the benefit reported in those with high parasite counts. Artesunate is a semisynthetic derivative of artemisinin whose water solubility facilitates absorption and provides an advantage over other artemisinins because it can be formulated as oral, rectal, intramuscular, and intravenous preparations. Artesunate is rapidly hydrolyzed to dihydroartemisinin, which is the most active schizonticidal metabolite. Injectable AS results in a more rapid systemic availability of AS compared with intramuscular AM. This pharmacokinetic advantage may provide a clinical advantage in the treatments of severe and complicated malaria.

## 1. Introduction

Malaria remains a tremendous health burden in tropical areas causing up to 24.3 billion episodes of clinical illness and 0.86 million deaths in 2009, with annual death rates of up to 93% of affected severe malaria [1]. A small proportion of children may also suffer from long-term neurological disability as a consequence of repeated bouts of severe malaria. Severe malaria occurs when infection with the *P. falciparum* parasite is complicated by serious organ failure or metabolic abnormalities; cerebral malaria, an unarousable coma not attributable to any other cause, is a specific type of severe malaria that even with appropriate treatment can have a mortality rate approaching 20% [1,2]. A small proportion of cerebral malaria survivors are left with persistent neurological sequelae. Severe malaria occurs most commonly in those with limited immunity to malaria. In highly endemic areas, young children are therefore at most risk of severe disease and death, whereas in areas of lower endemicity and travelers, both adults and children get severe disease [3]. There are currently three recommended treatments for severe and complicated malaria: artesunate (AS), artemether (AM) and quinine (or quinidine), although in many countries only quinine is available.

## 2. Limitation of Quinine in Treatments of Severe Malaria

Intravenous quinine, usually formulated as a dihydrochloride salt, is currently the most widely used agent in the treatment of severe falciparum malaria. In the USA, quinidine gluconate (the dextrorotatory optical diastereoisomer of quinine) is the only available intravenous antimalarial agent, and it may be used instead of quinine. The standard treatment for severe malaria is an intravenous infusion of quinine or quinidine [1,2]. Quinine may also be administered as an intramuscular injection. A loading dose of 20 mg/kg is recommended to reduce the time needed to reach effective concentrations in the blood [3]. A Cochrane Review found a significant reduction in fever clearance time and parasite clearance time with a loading dose compared with no loading dose, but concluded that data were insufficient to demonstrate an impact on risk of death [4].

For nearly 400 years quinine has been the principal drug used to treat severe malaria. Despite its long history of efficacy, quinine has significant limitations. Even with prompt administration, case-fatality rates in severe malaria often exceed 20%, especially, in areas of South East Asia [5]. Slow, constant intravenous infusion is the preferred route for giving quinine. This is not always possible and quinine can also be given by deep intramuscular injection into the anterior thigh. Intragluteal injection should be avoided because of the risk of sciatic nerve damage, and the absorption is slow and uncertain. A few studies have shown good efficacy and tolerability for rectal administration, without the problems of the intramuscular route or the complexity of intravenous administration [6].

Adverse effects resulting from quinine therapy are unfortunately common. Cinchonism often occurs at conventional dose regimens. This usually mild and reversible symptom complex consists of tinnitus, deafness, dizziness, and vomiting, and may affect patient adherence. Hypoglycaemia is a less common, but more serious, adverse effect. Some people are allergic to quinine and develop skin rashes and edema, even with small doses. Toxic levels of quinine can occur following rapid intravenous administration and can result in heart rhythm disturbances, blindness, coma, and even death; hence the recommendation for routine cardiac monitoring during parenteral treatment [5]. In accordance with the WHO policy of antimalarial drug usage in severe malaria treatment [1], quinine still remains the drug of choice in children when AS products are not available, although it suffers from certain drawbacks. Quinine has a narrow therapeutic ratio, causing hyperinsulinaemic hypoglycaemia (more frequent and severe in pregnancy) and prolongs the QTc interval when given parenterally, particularly if infused too rapidly. Intramuscular quinine is effective, but can cause local toxicity as well as hypoglycaemia in patients who may not have intravenous access [7]. Especially, in Southeast Asia there is evidence of increasing quinine resistance [8] so that artemisinins (mainly AS) are now increasingly used as first line treatments for severe malaria in many areas [2].

Quinidine is the only U.S. FDA approved drug for treatment of severe malaria. While it is commercially available and effective against malaria, it is not an ideal drug. Like quinine, quinidine is associated with sudden cardiac death, principally via cardiac arrhythmias, and, because of its short half-life, must be administered 2–3 times a day. Quinidine is more cardiotoxic than quinine and should be administered in an intensive care unit with continuous electrocardiographic and frequent blood pressure monitoring [9]. Quinidine-related cardiovascular adverse effects are potentially serious and may be more frequent if the drug is administered rapidly. The risk of cardiotoxicity is increased with bradycardia, hypokalemia, and hypomagnesemia and if the patient has received other drugs that may prolong the QTc interval (e.g., quinine, mefloquine, or macrolide antibiotics) [10]. 

## 3. Artemether Failed to Show Superiority over Quinine

Although still uncommon, resistance to quinine is being reported more and more often. Development of artemisinins for treatment of severe malaria has been conducted since 1987 [11]. The most important question in antimalarial drug treatment of severe malaria is whether the artemisinin derivatives can reduce mortality compared to quinine. Intramuscular AM has been the focus of many comparative studies versus parenteral quinine, with over 3,000 cases having been reported in Asia and Africa [12]. The large randomized comparisons of intramuscular AM and quinine in Gambian children [13] and Vietnamese adults [14] and a meta-analysis of individual data from 1,919 patients in 11 trials of parenteral therapy [5] identified no significant difference in efficacy between these agents using mortality as a primary end-point. However, in the meta-analysis, the subgroup analysis of adults had lower mortality when treated with AM. Initially, a number of trials of parenteral AM against quinine were undertaken in both children and adults with severe malaria [13,14,15]. In these trials, quinine was administered either intravenously or intramuscularly and AM was administered intramuscularly. AM is a lipid-soluble artemisinin derivative that can only be administered intramuscularly and is now known to be subject to more erratic uptake and a slower time to peak plasma concentrations than AS [16]. Although the results of these trials showed that AM was safe and easier to use than quinine, none of these trials showed a survival benefit individually (although there was the survival benefit demonstrated in the subgroup analysis of adults in Southeast Asia mentioned above) [5]. This evidence taken as a whole was not sufficiently convincing to prompt a policy change from quinine to AM.

Most significantly, as it turns out, AM may not have been the best choice of artemisinin derivative to advocate as a replacement to quinine, as the results of this study show that in terms of preventing deaths, AM and quinine are equal, a conclusion comparable to findings of the 11 trials in the meta-analysis [5]. Compared to AS, AM is less completely biotransformed to the more potent dihydroartemisinin and has slow, more erratic absorption after intramuscular administration; in fact, the ability of AM to provide equivalent benefit to quinine is probably testament to the antimalarial potency of the artemisinin derivatives as a group. Attention has therefore switched to AS.

## 4. Artesunate in Treatments of Severe Malaria and SEAQUAMAT Trials

Artesunate is a more attractive formulation than AM for pharmacokinetic reasons, as it can be administered either intravenously or intramuscularly, and it achieves therapeutic plasma concentrations rapidly when administered by either route [17]. Theoretically, therefore, AS may be more effective than AM, because AM is as effective as quinine. Initial trial results in adults were encouraging, with some evidence of survival benefits compared with quinine, albeit with small numbers [18]. For this reason the SEAQAMAT trial was undertaken between 2003 and 2005.

### SEAQUAMAT Trials

This was an open label, randomized, multicenter trial with centers in India (Rourkela, Orissa), Bangladesh (Chittagong), Myanmar (Myitkyina, Lashio, Taungoo, Tathon and Rangoon) and Indonesia (Timika). The large, randomized comparison of intravenous AS and quinine in 1,461 patients (including 202 children) in Asia showed a significant survival benefit with AS, 730 to the AS arm and 731 to the quinine arm. A total of 41 patients were subsequently excluded from the AS arm and 38 from the quinine arm owing to negative blood films for malaria parasites. Baseline characteristics and severity of disease were similar between the two groups. Mortality was 22% with quinine, as compared with 15% with AS, a risk reduction of 34.7% [19]. There was a large heterogeneity in overall mortality rates between countries with 28% in the Bangladeshi site compared with 9% in Indonesia. The mortality advantage of AS over quinine was preserved in all the study sites. The number needed to treat with AS to avoid one death ranged from 11.1 to 20.2 depending on the treatment site. Most of the mortality benefit occurred in the first few days of admission, with comparable mortality in both arms thereafter. The treatment effect was similar in children compared with adults but they constituted a much smaller group and results did not reach statistical significance [20]. Treatment with AS had a relatively mild side-effect profile; hypoglycemia was significantly more common with the use of quinine. A systematic review of five randomized trials comparing the efficacy of intravenous quinine with that of AS and one additional trial of intramuscular AS demonstrated the superiority of AS, with significant reductions in the risk of death (relative risk, 0.62), incidence of hypoglycemia, and parasite clearance time, as compared with quinine. Neurological sequelae were extremely rare, providing further reassurance of its safety. As a result of this study, parenteral AS is now recommended by WHO as the drug of choice for the treatment of severe malaria in low-transmission areas and in pregnancy in the second and third trimesters [21]. 

The current recommendation for treating severe malaria patients in high-transmission areas is either quinine or an artemisinin derivative. In these areas, severe malaria is mainly a pediatric disease, and in children mortality often occurs within the first 24 hours after admission, leaving a smaller time window for AS to deliver a clinical advantage. Additionally, African malaria parasites are more quinine-sensitive than those in Asia. Earlier studies comparing parenteral quinine with the oil-soluble derivative AM showed that AM was associated with a modest but significantly lower mortality than quinine in Asian adult patients, although this effect was not seen in African children [5]. Intramuscular AM has since been shown to be erratically absorbed in Vietnamese adults with severe malaria; it is unclear if this can explain the lack of effect on mortality in African children. Hence there is a need for an additional trial of the water-soluble AS in African children, which is currently underway.

The effective currently available AS formulation produced by the Guilin Pharmaceutical factory in China is manufactured to Chinese GMP standards, but is not ‘international GMP certified’. Further certification is under review. A new GMP formulation of AS is currently being developed by the Walter Reed Army Institute of Research (WRAIR), USA and should obtain FDA approval in the near future. Even if parenteral AS is widely available in hospitals, it will not be available to the majority of patients developing severe malaria living in rural tropics far from the nearest health clinic or hospital. For these patients, AS suppositories offer the prospect of effective early treatment, preventing clinical deterioration and buying time to reach hospital. The WHO has sponsored the development of GMP-manufactured rectal AS capsules, which are well absorbed and effective for the treatment of moderately severe malaria [22]. A large phase III study of early deployment has been completed and will be reported soon. Home- or village-based deployment of rectal AS in rural areas of Africa and Asia may play an important role in reducing malaria-associated morbidity and mortality.

Severe malaria remains a major global health problem and, despite advances in prevention, it is likely to remain so for the foreseeable future. The SEAQUAMAT study demonstrates convincingly that treatment of severe adult malaria acquired in Asia with AS is associated with a reduction in mortality compared with quinine. No ethical committee is likely to approve another similar study in adults with severe malaria in Asia and the SEAQUAMAT trial should be taken as definitive in this context. Can the results be assumed to also apply to children in Asia, and also to adults and children in Africa, where the bulk of severe falciparum malaria occurs? There are various reasons to be cautious about extrapolating the SEAQUAMAT data to all these groups. For adults with severe malaria, artesunate should be the treatment of choice. The drug is more effective than quinine, is simple to administer, and is safe. The only serious adverse effect that has been clearly linked with AS is a rare type-1 hypersensitivity reaction, which arises in about one in 3,000 treated patients. By contrast, quinine is locally toxic (particularly as the acidic dihydrochloride salt) after intramuscular injection (often causing sciatic nerve damage), cannot be given by bolus intravenous injection, requires three-times daily administration, is associated with potentially serious hypoglycaemia, and is less effective than AS. Unfortunately, despite 20 years of use in East Asia, there is still no generally available formulation of parenteral AS made to Good Manufacturing Practices standards. Quality assured and affordable parenteral AS should be made widely available in malaria endemic areas as a matter of urgency [22].

## 5. Advanced Effect of Artesunate Compared to Artemether and Quinine

Artesunate is a semisynthetic derivative of artemisinin whose water solubility facilitates absorption and provides an advantage over artemisinin because it can be formulated as oral, rectal, intramuscular, and intravenous preparations. Artesunate is rapidly hydrolyzed to dihydroartemisinin, which is the most active schizonticidal metabolite. Injectable administration of artesunate results in a more rapid systemic availability of AS compared with intramuscular AM. This pharmacokinetic advantage may provide a clinical advantage in the treatment of severe malaria [17]. Rectal AS has been shown to be absorbed rapidly, with a considerable inter-individual variability. Artesunate is highly effective against multidrug-resistant falciparum malaria and severe malaria in Vietnam, Thailand, China, and Myanmar [23]; however, only limited studies have been carried out in Africa.

### 5.1. Intravenous Artesunate in Replacement of Quinine for Adults and Pregnant Woman

As reported above, the South East Asian Quinine Artesunate Malaria Trial (SEAQUAMAT) Group published results of almost 1,500 patients with severe malaria showing that AS had superiority over quinine using mortality as an endpoint for the trial. This remarkable study added fuel to the fire that quinine, and in the U.S., quinidine, should be replaced by AS in the treatment of severe malaria. This study lead to the WHO’s guidelines at the end of 2005 and confirmed to the WRAIR development team that they had indeed made the right choice of drug candidate for development three years earlier. These results also provided encouragement for the team to redouble their efforts in getting this product to market as quickly as possible [17,24,25]. The problem to date had been that of getting a commercial partner. This drug, as noted earlier, generated little commercial interest because it was not patentable. Few pharmaceutical companies would be knocking at the door to share co-development costs with the Army in getting this product to market, much less take the chance of providing this drug over a sustained period after licensure. Luckily, there are new pharmaceutical companies emerging that specialize in niche markets such as orphan drugs. It was while the Army’s program was approaching its goal of putting together its filing of a New Drug Application with the FDA that WRAIR researchers met the subsidiary of an Italian drug company in the U.S., Sigma-Tau Pharmaceuticals Inc. that specializes in rare diseases and orphan drugs. This partnership was formed in 2009 and was upgraded to the parent company; Sigma-Tau SpA in 2010 so that this critically needed product was not only brought to the U.S. market, but also to the European and broader world market [25].

In pregnancy, the first trimester remains a concern with AS. Animal data have suggested an association of artemisinin drugs with fetal resorption and fetal abnormalities when administered early in pregnancy, although no increase in abortion or stillbirth rates were demonstrated in an observational study of 461 pregnant women on the Thailand–Myanmar border. The effects of AS in early pregnancy remain a concern in non-severe malaria but, since severe malaria is frequently associated with major adverse outcomes in pregnancy, the priority in treating a pregnant patient with malaria must be to administer an effective antimalarial quickly and, where quinine resistance is a possibility, this is likely to be AS. Rates of fetal death were similar in both treatment groups in the SEAQUAMAT trial, although only small numbers of patients were included and the outcomes of the surviving pregnancies were not reported. Balancing the potential risks of artemisinins to the fetus against the real risks of severe malaria to both of mother and fetus, artesunate should be recommended in all pregnant women from Asia with severe malaria. In pregnant women in their first trimester in Africa, the risks are more balanced; the potential teratogenicity remains the same while there is no proven advantage [20].

### 5.2. Intramuscular Artesunate in Replacement of Artemether or Quinine for Children

Intravenous AS and intramuscular AM have been highly efficacious for the treatment of severe malaria. Since AM failed to show superiority over quinine intravenously or intramuscularly [13,14,15,16], intramuscular AS is also effective and may be of value in settings with limited resources [15]. 

The intramuscular route for quinine administration has recently regained favor. It reduces the risk of dosing errors that can cause cardiotoxicity, although the risk of hypoglycemia is unaltered. For these reasons, there is an urgent need to develop alternative safe antimalarials that are efficacious in patients with severe malaria. Artemether is the most widely assessed of the parenteral artemisinin derivatives. Although it is an oil based formulation that can be given only by the intramuscular route, it has proved at least as effective as intramuscular quinine in the treatment of severe malaria in large randomized trials that have enrolled collectively nearly 2,000 patients both in Asia and in Africa [13,14,15]. Despite the generally good clinical results with AM, intramuscular AM only provides equivalent benefit to quinine [5]. Compared with AS, AM is less completely converted to the more potent dihydroartemisinin and has slow, erratic absorption after intramuscular administration; in fact the ability of AM is showing much less antimalarial potency when compared to AS [17]. 

Intramuscular AS has been used in trials with both uncomplicated and severe malaria patients [26,27,28]. Results showed when a child presents with severe malaria to a hospital in Africa, many factors influence the eventual outcome. These include both the rapidity of diagnosis and the speed with which effective treatment is given, especially as most deaths occur within the first 12 h after admission. At present there is no alternative therapy to quinine given in a loading dose by either the intramuscular route or the intravenous route. However, a clinical trial shows that intramuscular AS is an important alternative to quinine in Africa [28]. Also, intramuscular AS proved safe and effective in the treatment of severe and complicated childhood malaria in Asia [26]. 

The public health implications of suppository treatment of a life-threatening disease that kills millions of children in rural Africa each year are of great importance and warrant further clinical assessment of acceptability and implementation, although the possible consequences of widespread and relatively uncontrolled use of artemisinin and its derivatives also need to be assessed and taken into consideration.

### 5.3. Rectal Artesunate in Replacement of Quinine for Children

Many children in Africa die of malaria before reaching formal healthcare services. One attractive route of administration for AS in severe disease is the rectal route. Rectal AS is unlikely to challenge either parenteral AS or quinine in hospital practice, but it may have a significant role in pre-hospital settings, especially in rural parts of Africa where the bulk of malaria deaths occur. A trial in Uganda comparing rectal AM with intravenous quinine showed a trend towards faster parasite clearance and faster time to defervescence. However, these results failed to reach statistical significance to reduce death [29] and rectal AS may well fare better [22]. A recent multicenter trials of rectal AS used in a rural setting has recently been completed and had some mixed results when looking at the efficacy of high dose versus low dose, but the principal finding of increased survival in children using rectal AS who were not able to reach medical care within six hours of the diagnosis of malaria was well demonstrated [30]. 

Recent systematic reviews comparing rectal artemisinins with intravenous quinine in severe malaria have been published [30,31]. This review addresses the lack of any data directly comparing the therapeutic efficacy and safety of the different rectal preparations of artemisinin derivatives. The pooled analysis of individual patient data suggests that artemisinin and artesunate suppositories rapidly eliminate parasites and are safe. There is far less evidence for AM [29] and no studies of dihydroartemisinin suppositories were available to be included in this analysis. The results indicate that both artemisinin and AS, whether as single or multiple dose regimens, induce a superior parasitological response than parenteral quinine over the 24 hours following initiation of treatment. Regimens employing a higher single dose of rectal AS were five times more likely to result in >90% parasite reductions at 24 hours than were multiple lower doses of rectal AS or than a single administration of AM. These results imply that dosage regimens that result in immediate high blood concentrations of drug [22,32] are those best able to reduce parasitemia in patients with evolving severe malaria and that sustained drug exposure achieved by sequential treatment with moderate doses offers no therapeutic advantage [33].

The SEAQUAMAT trial comparing intravenous AS with parenteral quinine demonstrated a 35% lower mortality with AS, confirming that more rapid initial parasite clearance may translate to reduced mortality in severe adult malaria [19]. Therefore, although the current pooled analysis was not powered to assess mortality as an endpoint, the differences in parasite clearance rates between rectal artemisinins and parenteral quinine, and between different rectal artemisinin dosing regimens, should be regarded as important indicators of possible real differences in therapeutic efficacy and clinical benefit. It should also be noted that any future study designed to use mortality as an endpoint to compare different rectal artemisinins would require such a large sample size that it is unlikely ever to be implemented. Therefore the surrogate marker of parasite clearance used in this analysis is likely to remain the best available evidence on which to base comparisons of treatment efficacy for the rectal artemisinins.

The evidence from this analysis shows rectal AS has superior effect in reducing parasite densities compared to quinine (intravenous or intramuscular) at 12 and 24 hours after administration. The result supports the WHO recommendation for the use of AS and artemisinin as initial pre-referral treatment [21]. Basis of systematic review, the WHO recommendation is to use artemisinins rectally for complete treatment only when parenteral antimalarial treatment is not possible [30,31].

### 5.4. Artesunate Is the Best Choice for the Severe and Complicated Malaria Therapy

These trials conducted in intravenous, intramuscular and rectal routes with AS and compared to AM and quinine demonstrated that AS should now become the therapy of choice for the severe malaria, undoubtedly in adults, although more information may be needed in children living in high transmission settings [19,34,35,36]. Severe malaria is a medical emergency. After rapid clinical assessment and confirmation of the diagnosis, full doses of parenteral antimalarial treatment should be started without delay with whichever effective antimalarial is first available. Recently, WHO recommended that AS should be in the first selection for treatment of severe malaria. 

Artesunate given at 2.4 mg/kg bodyweight intravenous (i.v.) or intramuscular (i.m.) on admission (time = 0), then at 12 hours and 24 hours, then once a day, is the recommended choice in low transmission areas or outside malaria endemic areas. For children in high transmission areas, the following antimalarial medicines are recommended as there is insufficient evidence to recommend any of these antimalarial medicines over another for severe malaria:

- AS 2.4 mg/kg bodyweight i.v. or i.m. given on admission (time = 0), then at 12 hour and 24 hour, then once a day;
- AM 3.2 mg/kg bodyweight i.m. given on admission then 1.6 mg/kg bodyweight per day;
- quinine 20 mg salt/kg bodyweight on admission (i.v. infusion or divided i.m. injection), then 10 mg/kg bodyweight every 8 hours; infusion rate should not exceed 5 mg salt/kg bodyweight per hour.

A major concern that has been expressed is that the current production of AS does not meet Good Manufacturing Practice (GMP) standards, although there is no evidence that this translates into reduced efficacy. This is likely to be a temporary issue, although these concerns may give rise to delayed uptake in some settings, especially in treating patients from Africa where the evidence is less certain. GMP products are currently under development, both in China and in a collaboration involving the Walter Reed Army Institute of Research (WRAIR) [25].
